# Language learning in the adult brain: disrupting the dorsolateral prefrontal cortex facilitates word-form learning

**DOI:** 10.1038/s41598-017-14547-x

**Published:** 2017-10-25

**Authors:** Eleonore H. M. Smalle, Muriel Panouilleres, Arnaud Szmalec, Riikka Möttönen

**Affiliations:** 1 Psychological Sciences Research Institute, Université catholique de Louvain, Louvain-la-Neuve, Belgium; 2 Institute of Neuroscience, Université catholique de Louvain, Louvain-la-Neuve, Belgium; 3 Department of Experimental Psychology, University of Oxford, Oxford, United Kingdom; 4 Department of Experimental Psychology, Ghent University, Ghent, Belgium; 5 School of Psychology, University of Nottingham, Nottingham, United Kingdom

## Abstract

Adults do not learn languages as easily as children do. It has been hypothesized that the late-developing prefrontal cortex that supports executive functions competes with procedural learning mechanisms that are important for language learning. To address this hypothesis, we tested whether a temporary neural disruption of the left Dorsolateral Prefrontal Cortex (DLPFC) can improve implicit, procedural learning of word-forms in adults. Young adults were presented with repeating audio-visual sequences of syllables for immediate serial recall in a Hebb repetition learning task that simulates word-form learning. Inhibitory theta-burst Transcranial Magnetic Stimulation was applied to the left DLPFC or to the control site before the Hebb task. The DLPFC-disrupted group showed enhanced learning of the novel phonological sequences relative to the control group. Moreover, learning was negatively correlated with executive functions that rely on the DLPFC in the control group, but not in the DLPFC-disrupted group. The results support the hypothesis that a mature prefrontal cortex competes with implicit learning of word-forms. The findings provide new insight into the competition between brain mechanisms that contribute to language learning in the adult brain.

## Introduction

It is well known that children surpass adults in their language learning ability, in particular for certain aspects of language that involve grammar and phonology^1,2^, but it has remained unclear why this is the case^3,4^. Adults outperform children in most measures of cognition, especially those that rely on the prefrontal cortex that maturates until adulthood, such as executive functions, attention and working memory^5^. Yet, they fail learning languages with the ease that children do. It has been proposed that the mature brain systems supporting these cognitive functions interfere with implicit procedural learning, which contributes to certain aspects of language learning such as word-forms or grammar^6–9^. However, there is little experimental evidence supporting this hypothesis.

Multiple brain systems support learning in cooperative and sometimes competitive ways^10,11^. For example, procedural and declarative memory systems are known to interact during learning^12^. The declarative memory system is characterized by voluntary processes that rely on attentional resources mediated by prefrontal and medial-temporal lobe structures. Procedural memory on the other hand is part of implicit memory. Learning in implicit memory takes place without the intention to do so, and so whereby awareness of the process or the outcome is not needed for learning to occur^13,14^. Procedural memory relies on such learning allowing the acquisition of a sequence through repeated exposure. In this paper, we use the terms “implicit learning” and “procedural learning (of sequential information)” interchangeably. This form of implicit memory is mainly mediated by striatal structures (see ref.^15^), however, it also interacts with other networks, such as the medial-temporal lobe^16^ and parts of the prefrontal network^15,17,18^. Procedural memory plays an important role in automatic skill learning such as motor learning (e.g., riding a bicycle) as well as certain aspects of language learning, in particular for grammar, phonology or word-forms that have sequential structures^8,19,20^. Studies on motor learning suggest that suppression of prefrontal activity, due to hypnosis^21^, repetitive Transcranial Magnetic Stimulation (TMS)^22^, use of secondary or distraction tasks^12,23^, cognitive fatigue^24^, alcohol consumption^25^, or intake of benzodiazepines^26^, can enhance procedural learning of a novel skill. Moreover, motor learning studies have shown that enhancing cognitive effort during learning results in impaired procedural learning^27^ with greater activity in the prefrontal cortex^28^. These studies support the idea that the suppressed reliance on the prefrontal lobe, and in particular conscious executive functions that support declarative memory, improves implicit procedural learning of a novel skill.

A hypothesis of competing cognitive and procedural mechanisms during learning is also gaining attention in the field of language e.g., refs^6,7,20^. For instance, learning a novel word involves multiple cognitive processes, including segmentation abilities as well as retention of sequences of constituent phonemes or syllables and creation of long-term phonology-to-semantic connections^29,30^. The long-term memorization of phonological sequences, or word-forms, is argued to rely on procedural memory mechanisms^8^. Experimental evidence for this comes from research with the *Hebb repetition paradigm* ^31–33^. In this task, sequences of items (e.g., phonemes/syllables) have to be retained in short-term memory for immediate serial recall. One sequence (often called the Hebb sequence) is repeated in exactly the same order every n^th^ trial but this is not told to the participants. Recall performance for the Hebb sequence usually improves across trials relative to the non-repeating (filler) sequences. An increasing amount of experimental work is consistent with the hypothesis that the mechanisms underlying the Hebb repetition effect (HRE) are related to word-form learning, in particular to the consolidation of novel word-forms in memory. Indeed, syllable sequences acquired through Hebb repetition learning compete with existing word-forms in the lexicon indicating that the learned sequences are integrated in long-term memory as novel lexical forms^34–36^. Learning in the task is also fast and long-lasting^37^, and is found to correlate with measures of vocabulary development in young children^38,39^. Similarly to novel word-form learning, the occurrence of the HRE does not rely on explicit learning mechanisms (i.e., the intention to learn)^40^. For instance, prior awareness of the repeating sequence or explicit reproduction during the recall phase does not lead to better learning^41–43^. Also, focal hippocampal lesions do not affect the HRE^44^. Therefore, Hebb repetition learning is argued to be implicit in nature^13,14,40^.

Recent developmental studies show that eight–year-old and eleven-year-old children outperform adults in the implicit learning of novel word-forms^45,46^. Interestingly, in the Hebb-learning study of Smalle *et al*.^40^, the age-effect depends on the item-overlap between Hebb and filler sequences. When the phonological Hebb sequences contain different syllables as used in the random filler sequences (so-called non-overlapping sequences), the HRE is greater in children than in adults. This child advantage is absent and the HRE decreases in both adults and children, when the phonological Hebb sequences contain the same syllables as used in the random filler sequences, but in a different order (so-called overlapping sequences). It is likely that the item-overlap with the filler sequences interferes with the sequence learning processes that are necessary for the long-term memorization of the word-forms in the Hebb task^37,45^. When adults are forced to attend to smaller two-syllable-structures within the sequence (thereby simulating children’s smaller working memory capacity), the HRE for non-overlapping, but not overlapping, sequences increases to the same level as children^45^. These findings suggest that children outperform adults in procedural language learning tasks that benefit from limited capacity in working memory.

The aim of the present TMS study was to determine whether neural inhibition of the dorsolateral prefrontal cortex (DLPFC) that supports executive functions and declarative memory, facilitates learning of word-forms in adults. Participants received disruptive repetitive TMS over the left DLPFC or a control site just before performing the Hebb repetition learning task. We hypothesized that TMS-induced disruption of DLPFC should increase the HRE for the non-overlapping Hebb sequences (but not for the overlapping Hebb sequences^45^) if the DLPFC interferes with implicit learning of novel phonological forms in adults. We also predicted that the individual differences in performance in additional executive function tasks (from now on referred to as EF tasks) would correlate with individual differences in the HRE measured in the control group, i.e. in the absence of TMS-induced disruption of the DLPFC. Based on this prediction, we expect that, in the control group, participants with high performance in EF tasks would show a decreased HRE relative to participants with lower performance in EF tasks. We also predicted that individual differences in EF performance would not correlate with the HRE measured during TMS-induced disruption of the DLPFC, because the stimulation was expected to decrease the interference of the DLPFC and increase the HRE.

## Results

### DLPFC disruption specifically enhances the learning of non-overlapping sequences

**Figure 1:**
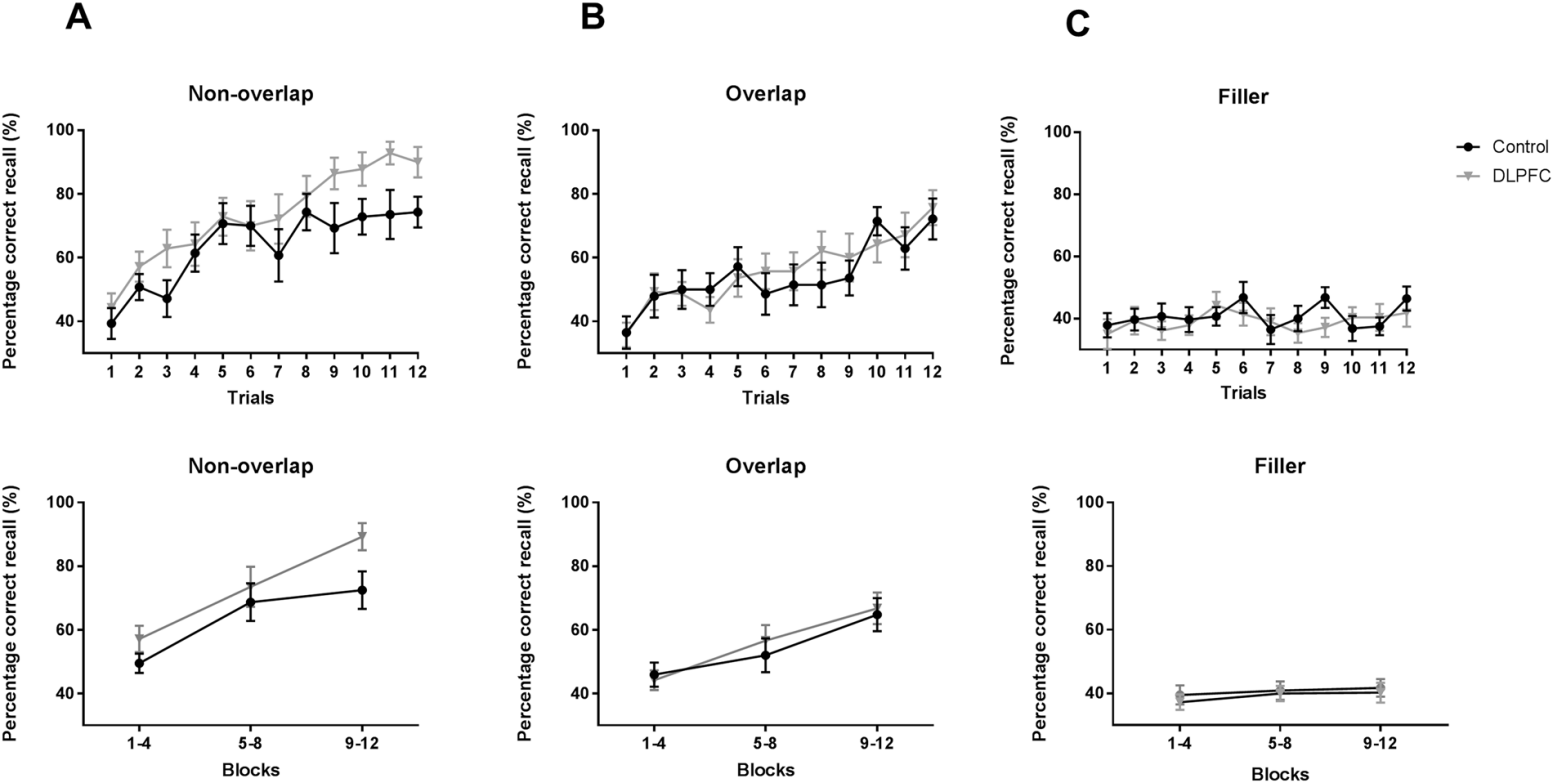
The effect of TMS-induced disruption of DLPFC on Hebb learning. Sequence learning across trials (upper panel) or block of trials (lower panel) is plotted for (**A**) the non-overlapping Hebb trials, (**B**) the overlapping Hebb trials, and (**C**) the filler trials, after repetitive TMS over the left DLPFC (n = 14) and control site (n = 14). Percentage correct recall of the non-overlapping Hebb sequence was higher in the DLPFC-disrupted group than in the control group during the last block of trials. Error bars represent standard error of the mean.

The immediate-recall performance for the non-overlapping and overlapping Hebb sequences and the filler sequences is presented in Fig. 1 for the control group (N = 14) and the DLPFC group (N = 14). There was a significant interaction between Sequence Type (3) and Block (3) (*F*_4,104_ > 19.8, *P* < 0.001, *n*_p_^2^ > 0.43). Separate ANOVAs for each Sequence Type showed that the recall accuracy increased across the three blocks for non-overlapping (main effect of Block: *F*_2,52_ > 41.2, *P* < 0.001, *n*_p_^2^ > 0.77) and overlapping (main effect of Block: *F*_2,52_ > 20.9, *P* < 0.001, *n*_p_^2^ > 0.63) Hebb sequences but not for the Filler sequences (no significant main effect of Block). These improvements in performance show that participants were able to learn both overlapping and non-overlapping Hebb sequences. However, the improvement in performance was greater for non-overlapping sequences than for overlapping sequences (i.e., Sequence Type (2) × Block (3), *F*_1,26_ > 14.41, *P* < 0.001, *n*_p_^2^ > 0.36). TMS-induced disruption of DLPFC modulated learning (significant Sequence Type (3) × Block (3) × TMS Group (2) interaction: *F*_4,104_ > 2.88, *P* < 0.05, *n*_p_^2^ > 0.10). More specifically, separate ANOVAs for each Sequence Type showed that performance for the non-overlapping sequence was enhanced in the DLPFC group compared with the control group in the last block of trials (main effect of TMS Group, *F*_1,26_ > 6.5, *P* < 0.05, *n*_p_^2^ > 0.20; and a Block (3) × TMS Group (2) interaction: *F*_2,52_ > 4.7, *P* < 0.05, *n*_p_^2^ > 0.15). No difference between groups was found for the overlapping Hebb sequence or for the filler sequence. These findings support our hypothesis that the DLPFC interferes with Hebb learning.

### Correlations between Hebb learning and executive functions

To further explore the relationship between Hebb learning and executive functions, we ran correlational analyses for the control (n = 14) and the DLPFC-disrupted (n = 14) groups separately. There were no significant differences between the two groups in the composite scores for the EF tasks, measured in a separate follow-up session (DLPFC group, *M*_*EF*_ *= *−0.001, SD = 1.5; Control group, *M*_*EF*_ = 0.002, SD = 1.2; *F* < 1).

**Figure 2:**
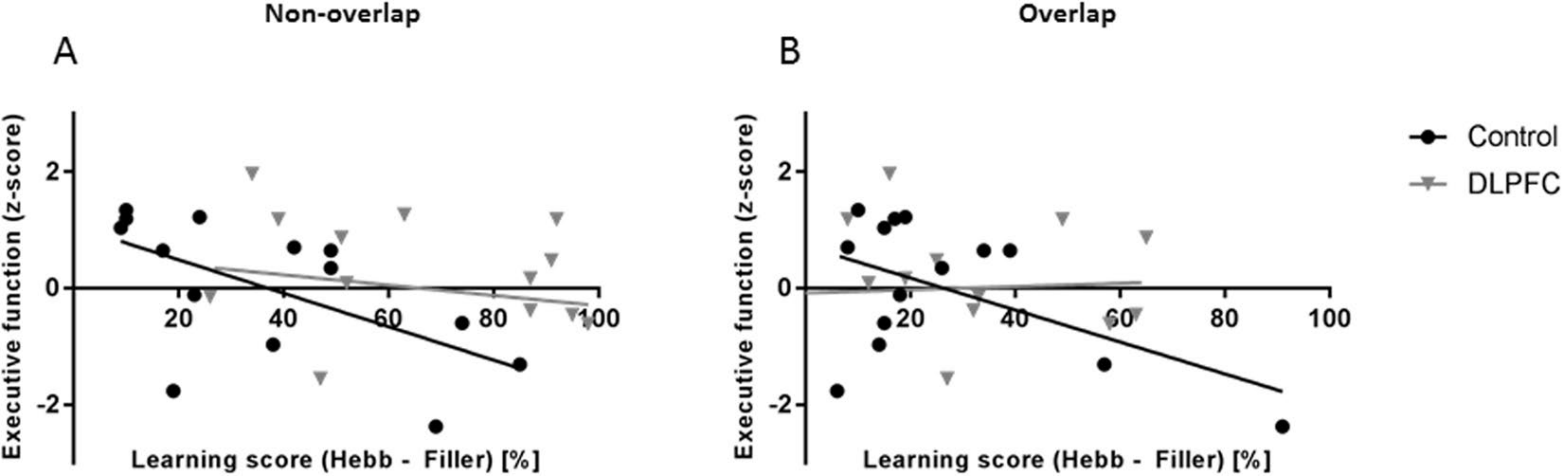
Relationship between Hebb learning and executive functions. HRE for non-overlapping sequences (**A**) and overlapping sequences (**B**), and an index of executive functions is plotted for each participant. (**A**) HRE for non-overlapping sequences correlated significantly with executive functions in the control group (n = 14, black circles), but not in the DLPFC-disrupted group (n = 14, grey triangles). (**B**) HRE for overlapping sequences did not correlate with executive functions in either group.

The Hebb learning score for the non-overlapping sequences showed a significant negative correlation with executive functions in the control group (r_(14)_ = −0.601, *P* = 0.023, Fig. 2 A), but no significant correlation in the DLPFC-disrupted group (r_(14)_ = −0.149, *P* = 0.612, Fig. 2 A). In addition, we ran further partial correlation analyses between Hebb learning and executive functions controlling for phonological working memory (measured by the digit span task)^25^ and found again a negative correlation between Hebb learning and executive functions in the control group (r_(11)_ = −0.613, *P* = 0.026), but not in the DLPFC-disrupted group (r_(11)_ = −0.174, *P* = 0.570). These results give further support for our hypothesis that, in the non-disrupted condition (control group), executive functions interfere with Hebb-learning.

The partial correlations between Hebb learning scores for overlapping sequences and executive functions (controlling for phonological working memory) were non-significant in both groups (DLPFC: r_(11)_ = 0.088, *P* = 0.776; Control: r_(11)_ = −0.513, *P* = .073; Fig. 2 B). The close to significant trend in the control group was caused by one participant, who had a large HRE for overlapping sequences (partial correlation without this outlier: r_(10)_ = −0.151, *P* = 0.640).

### Post-learning awareness

Participants gained a moderate awareness for the repetitive occurrence of the two Hebb sequences (question 1, DLPFC, *M*rating = 3.2, SD = 0.80; control, *M*rating = 3.4, SD = 0.74) and a weak awareness for the overlapping nature between one of the Hebb sequences and the filler sequences (question 2: DLPFC, *M*rating = 2.2, SD = 0.89; control, *M*rating = 2.2, SD = 0.70). There was no difference in awareness between the two groups (question 1, *Z = *−0.481, *P* = 0.630; question 2, *Z = *−0.099, *P* = 0.921) suggesting that the increased learning performance for the non-overlapping Hebb sequence of the DLPFC group is not due to a difference in awareness of the sequence repetition.

## Discussion

We addressed the hypothesis that the left dorsolateral prefrontal cortex competes with procedural learning mechanisms that are important for language learning, potentially explaining the inferior language learning skills in adults relative to children. We used TMS to examine whether neural inhibition of the left DLPFC would improve the implicit sequence learning that underlies word-form learning in adults. We found that the group with disrupted DLPFC showed improved Hebb repetition learning compared to the control group, but only for sequences that did not overlap with the filler sequences. This supports our hypothesis that in the adult brain, executive functions, supported by the DLPFC, compete with word-form learning. Moreover, we found a negative relationship between Hebb learning and performance in additional EF tasks in control participants – such that participants with higher scores on these tasks showed reduced Hebb repetition learning for the non-overlapping Hebb sequences compared to participants with lower scores.

Overall, our findings are in line with previous studies demonstrating that reducing the reliance on the prefrontal cortex can improve task performance^15,17,21,22,25^. For example, Udden and colleagues found that disruptive TMS to the left ventral lateral prefrontal cortex (VLPFC) improves syntactic classification performance following artificial grammar learning^17^. Moreover, Galea and colleagues found that disrupting the DLPFC using TMS enhanced performance in the serial reaction times task, which is a well-known procedural motor learning task^22^. The current study tested, for the first time, whether disrupting the DLPFC, similarly enhances performance on the Hebb repetition paradigm that links procedural sequence learning directly to word learning. Procedural memory is thought to be important for certain aspects of language learning, such as grammar and word-forms, while other more idiosyncratic aspects, such as associating semantics with phonology, rely more on an explicit declarative memory system. In a recent study, Finn and colleagues demonstrated that adults’ cognitive functions that involve attention and effort interact with specific language-learning processes^6^. For instance, directing effort or attentional control toward the phonological input benefits word-segmentation but disrupts learning phonological categories of a novel word-form structure. The authors argued that competition from executive functions, such as effort or the allocation of attention that support a late-developing declarative memory system, can explain why adults have difficulties in some but not all aspects of language learning compared to children, especially in the aspects that are related to procedural memory. In a previous Hebb learning study, we showed that children outperform adults in the Hebb repetition learning of phonological sequences that resemble novel word-forms^45^. However, when adults were forced to attend to the small parts of the sequence instead of the entire sequence as a whole, thereby simulating children’s limited working memory capacity, Hebb learning performance for the non-overlapping sequences increased to the same level as in children. The findings of the current TMS study extend these behavioral findings by providing direct evidence for the disruptive role of the DLPFC, the brain region related to the conscious executive processes in working memory and assumed to develop late across childhood, during Hebb learning in adults. Together with earlier studies on motor learning, the current results support the idea that different memory systems compete during automatic skill learning^12,47,48^.

The HRE was smaller for the overlapping sequences than for the non-overlapping sequences, and TMS-induced disruption of the DLPFC modulated Hebb learning performance for the non-overlapping sequences, but not for the overlapping sequences. The overlap with the filler sequences therefore seems to counteract the sequential learning processes that underlie Hebb repetition learning. In an fMRI study, Kalm and colleagues showed that activity in the medial-temporal lobe is correlated with learning overlapping sequences in a Hebb repetition paradigm^16^. Although no comparison was done with non-overlapping sequences, this may suggest that item-overlap potentially recruits different, declarative-based memory resources, or more attentional control, to deal with the proactive interference between sequences. It could explain why disrupting the DLPFC did not yield the same beneficial effect on overlapping sequences as it did on non-overlapping sequences. Further research is needed to explore what role memory and other cognitive processes play in word-form learning, and how this is modulated by item-overlap or interference between sequences.

Correlation analyses showed that performance in the EF tasks, i.e. the Winsconsin Card Sorting Task and the Semantic Fluency Task, was negatively correlated with learning the non-overlapping Hebb sequences in the control group only. This supports our hypothesis that executive functions that are supported by the prefrontal cortex interfere with sequential processes related to word-form learning. The lack of a significant correlation in the DLPFC-disrupted group provides further support for this hypothesis. In this group, Hebb learning was measured during TMS-induced disruption of the DLPFC, whereas EFs were measured in the absence of DLPFC disruption. The disruption decreased the interference of DLPFC, which supports EFs, on Hebb learning. Consequently, the individual Hebb learning scores were close to the ceiling (i.e., 100%) in the final block of trials in this group, and this did not correlate with performance in EF tasks tested in a subsequent session. Although the negative correlation between executive functions and sequence learning in the control participants is in line with a number of previous studies e.g., ref.^25^, it is not a consistent finding^49,50^. Some studies found no relationship between executive functions (or related working memory processes) and sequence learning e.g., ref.^28^ and others even found a positive (supporting) relationship^18^. Working memory is a complex construct that is composed of several components (i.e., domain-specific stores and an executive control system) that are operationalized by different tasks (e.g., span tasks vs. change detection tasks)^51^. The use of different tasks could have lead towards inconsistent outcomes in previous studies^50^. In a Hebb-learning task, participants must retain a sequence of syllables in short-term memory for immediate serial recall, suggesting a supportive role of working memory processes in sequence learning. This does not suggest, however, that all working memory processes, such as executive functions, support implicit learning. More research is needed to understand the complex relationship between working memory and implicit sequence learning across development^49^.

Here, we found enhanced learning of non-overlapping verbal sequences following disruptive TMS over the left DLPFC. This finding can be interpreted in line with our hypothesis that DLPFC competes with the procedural memory system in the adult brain. A potential alternative explanation for our finding is that TMS over DLPFC changed the manner of processing (e.g., speed of processing) in this region or in a connected region, resulting in more efficient procedural learning of word-forms. This alternative explanation cannot be ruled out. Since the continuous theta-burst stimulation (cTBS) that was used in our study has been shown to inhibit the functioning of the targeted motor areas^52,53^, we believe that it is likely that cTBS inhibited the left DLPFC and this inhibition improved performance in the (Hebb) learning task^22,54^. The negative correlation between executive functions and learning is also in line with the hypothesis that prefrontal regions interfere with procedural learning. Future studies are however needed to investigate the role of DLPFC in learning of verbal sequences.

In summary, our findings demonstrate that TMS-induced disruption of the left DLPFC improves word-form learning in young adults. These findings are in line with earlier studies, which found a similar beneficial effect of suppressed prefrontal-dependent functions on procedural learning^15,17,21,22,25^. The current findings build on developmental studies with the Hebb paradigm, showing that adults do not learn Hebb sequences as easily as children do^45^. Procedural memory maturates early in development and is important for skill acquisition and language learning^20^. In contrast, the prefrontal-dependent executive functions and declarative memory mature late in development^5^. To conclude, the results clearly show that DLPFC is causally involved in Hebb repetition learning in adults. Our interpretation is that a mature DLPFC interferes with procedural learning of non-overlapping syllable sequences. We believe that this can explain differences between adults and children in learning some aspects of language (e.g., novel word-forms). Competition between brain systems may offer a domain-general mechanism for understanding maturational sensitivities in acquisition of various skills, including language.

## Methods

### Participants

Thirty participants were recruited in the present study. One male participant was excluded from further analysis because he performed more than 2 standard deviations (SD) below mean on overall recall accuracy in the learning task. One female participant was excluded as she performed more than 2 SD below mean for performance on the executive function tasks. Hence we report the data of 28 participants, who were randomly assigned to the left DLPFC group (n = 14, 10 females; mean age: 23.9 SD: 2.8) and the control group (n = 14, 8 females; mean age: 22.8; SD: 2.4). All participants gave their written informed consent prior to the study and were blind to the purpose of the study. Participants were right-handed, with no hearing or language impairment and no neurological conditions. They were all native English speakers except for two (one in each group) who were fluent English speakers (native German). Participants received financial compensation at the end of the experiment (£10/hour). The procedure was approved by the Central University Research Ethics Committee (CUREC) at the University of Oxford (Reference: R45415/RE001).

### Experimental Design

Participants were familiarized with the Hebb learning task before receiving the continuous Theta Burst Stimulation (cTBS) to the left DLPFC or the control site. cTBS was immediately followed by the Hebb learning task, which lasted approximately 30 min. During a follow-up session that took place five to six hours later, two EF tasks and one verbal short-term memory task were administered. The administration took place in a separate session in order to avoid possible confounding effects between the cognitive tasks and the stimulation. After the completion of the experiment, participants filled in a post-learning awareness questionnaire in which they were asked to report their knowledge about the unannounced sequence repetitions.

### Continuous Theta Burst Stimulation (cTBS)

TMS was delivered using a 70-mm diameter figure-eight coil (Rapid^2^ stimulator; Magstim, Whitland, UK). The control site was located 2 cm posterior to the vertex while the DLPFC was localized using the BeamF3 algorithm^55,56^. For each participant, the left motor cortex was identified as the spot eliciting reliable twitches in the resting contralateral hand. The active motor threshold (aMT) was defined as the lowest intensity at which TMS elicited at least five out of ten visible muscle twitches, whilst the subject sustained a light contraction of their pinch (index finger and thumb). There were no significant differences in aMT between groups (i.e. DLPFC group, *M* = 57.5%, SD = 8.2; Control group, *M* = 57.0%, SD = 6.5; *F* < 1). The intensity of the stimulation was set at 80% of the aMT. The coil was placed tangentially to the scalp with the handle pointing posterior at a 45° angle with respect to the anterior-posterior axis for DLPFC and at 0° for the control site. A modified cTBS protocol was used in which 600 pulses were delivered in a continuous train of 200 bursts. Each burst consisted of 3 pulses at 30 Hz, repeated at 6 Hz. This modified cTBS protocol has been shown to inhibit cortical excitability lasting at least 30 minutes after stimulation over the primary motor cortex^52^.

### Tasks

#### The Hebb repetition task

The Hebb repetition task was composed of 48 trials. During each trial, ten consonant-vowel (CV) syllables were presented to the participants for immediate serial recall. Participants were, in total, exposed to three different types of trials: the filler sequence (24 trials), the Hebb sequence with CV syllables overlapping with the filler sequence (12 trials) and the Hebb sequence with CV syllables non-overlapping with the filler sequence (12 trials). Two pools of 20 unique CV’s were created (Table 1). Half of the participants in each group were exposed to the two first CV pool while the other half was exposed to the second CV pool. From each pool, two sequences of 10 serially ordered CV’s were created to represent the two Hebb sequences (Table 1), and matched for summed bigram frequencies as measured within the speaker’s native language^57^; (i.e., all *p*s > 0.05). The filler sequences were composed of the same CV’s as one of the two sequences but in a random order. This order changed every trial.

**Table 1:** The sequences that were presented during the Hebb learning task.

	CV1	CV2	CV3	CV4	CV5	CV6	CV7	CV8	CV9	CV10
Pool 1	SE	FU	BE	DI	ZA	RU	GU	MI	TU	VY
	MA	PO	FE	BY	HY	SA	FI	TA	RE	JI
Pool 2	HY	RU	FE	FU	ZA	SA	RE	MA	FI	MO
	TU	DI	ME	JI	VI	RI	SE	PO	GU	VY

A trial consisted of the successive presentation of the ten CV syllables both in their auditory and written forms for 500 ms each with an inter-stimulus interval of 388 ms. All syllables were recorded by a female native British-speaker. They were presented auditory at 60 dB using Sennheizer HD201 headphones and appeared visually using Courrier New letter type with point size 24. Immediately after presentation of the ten syllables, a recall screen was presented on the screen with all ten syllables randomly organized in a circle around a central question mark. The participants were asked to click the syllables using the mouse in the order the syllables were presented. To maximize free recall of the sequences, participants did not receive feedback about their performance. Prior to cTBS, participants were familiarized with the task by performing two filler sequences trials (that were derived from the other pool). After cTBS, the task continued with a filler sequence, one of the two Hebb sequences, another filler sequence and the other Hebb sequence. Hence, the two Hebb sequences, the one overlapping and the one not overlapping with the filler sequences, were mixed within the same task and were repeated every fourth trial. The order of the type of Hebb sequence (overlapping first or non-overlapping first) within the task was counterbalanced across participants. In total, the Hebb repetition learning task lasted for ~20 minutes. This kind of design, mixing overlapping and non-overlapping sequences, does not affect the occurrence of a HRE^45,58^.

#### Digit span

The digit span task is a measure of phonological storage capacity in working memory^59^. In this task, participants listen to an experimenter reading sequences of digits of increasing length and repeat them forward or backward. First the forward digit span was administered followed by the backward one. Starting with two-item sequences, a maximum of two trials was presented at each length. If one of the two trials at a particular sequence length was correctly repeated, the sequence length increased by one. The task stops when the participant fails to repeat two sequences of the same length. The digit span refers to the maximum length that the participant can repeat successfully. The forward and backward digit spans were summed to obtain one global score for the digit span task.

#### Winsconsin Card Sorting Task (WCST)

WCST is one of the most well-known measures of EFs, assessing cognitive flexibility and inhibitory control in working memory^60,61^. In this task, participants are required to derive a correct card-sorting rule (e.g., cards are sorted as a function of color similarity) based on a trial-by-trial feedback. As the rule changes without announcement (e.g. the cards are sorted as a function of shape similarity), the participant has to detect the novel rule and modify the previously learned response strategy based on the feedback he receives from the experimenter. The key measure is the number of perseveration errors that is counted when the participant persists in using the old rule despite negative feedback. The lower the number of perseveration counts, the better the EF performance. The test was run using the online software package PsyToolKit^62,63^.

#### Semantic Fluency Task

The semantic fluency task is another widely used task to measure EF sevaluating lexical-access ability from declarative memory^64^. In this task, participants are instructed to produce as many words belonging to the same category (i.e. animals) as possible in 60 sec, without repetitions, synonyms, or altered forms of the same word^65^. Higher scores (i.e. more words) reflect better EF.

### Post-learning awareness

At the end of the experiment, a pencil-and-paper awareness report concerning explicit knowledge about the repeating contents of the Hebb task was administered. This questionnaire included two questions in the following order based on^42,66^: “*Did you notice that there were two sequences in which the same order was repeated every fourth trial*?”; and “*Did you also notice that one of the two sequences that were repeated every fourth trial contained the same syllables as the other nonrepeating sequences?”* On each question, the participant rated his/her awareness with the following four-point scale: 1) No experience: I had no impression during the whole duration of the task; 2) Weak experience: I had a feeling that “something” was occurring, but I would not have been able to specify myself what is was; 3) Almost clear experience: I sometimes had the impression but I was not always sure. Now- because you say so – I am sure there is.; 4) Clear experience: I already had this impression clearly during the course of the task, no doubt. The use of verbal reports after learning is a common procedure and a relatively sensitive measure for demarcating task awareness in humans^14,67^.

### Statistical Analysis

The Hebb learning performance was scored using McKelvie’s method (1987) implemented into a script running in Python (2.6+). This scoring method takes into account both the position and the serial order of correctly recalled items. In a first step, the number of syllables that are in the correct position from left to right is counted up to the first error. Secondly, the same step is repeated from right to left up to the first error. After this, the number of items in any correct sequence of two or more items between the first error from the left and the first error from the right is counted. Finally, any other items that occur in the correct absolute position from left to right are counted. The maximum possible score using this method is ten (the entire length of the sequence). Recall scores for the filler sequences were averaged across two consecutive filler trials to obtain an equal number of filler and Hebb scores. All correct trial scores were subsequently transformed into percentage scores (Fig. 1 upper panel) and organized into three blocks of 4 trials for analysis (Fig. 1 lower panel).

Statistical analyses were performed with the SPSS Statistics software package (IBM, Armonk NY, USA). An arcsine square root transformation was completed on the percent scores in order to reduce skew and make the distribution appropriate for statistical analyses^45,68^. In order to test whether TMS-induced disruption affected Hebb learning, we analyzed the transformed trial scores using a mixed design ANOVA with Sequence Type (3: overlapping Hebb, non-overlapping Hebb vs. Filler) and Block (3) as within-subject factors and with TMS Group (2: DLPFC vs. Control) as between-subject factor. In order to interpret significant interactions, we run separate ANOVAs for each sequence type. Planned comparisons were conducted using Fisher’s LSD tests.

In order to further investigate the relationship between EF and Hebb learning, we calculated a composite score for EFs by transforming measures of fluency task and WCST into positive z-scores and summing them for each participant as in Nemeth *et al*. (2013). We then tested whether these composite scores for EF correlated with Hebb learning scores (calculated as the difference between the Hebb and Filler scores in the last block of trials). Awareness scores from the post-awareness questionnaire were also compared between groups using non-parametric Wilcoxon tests. All significant results (p < 0.05) are reported together with the *η* ^2^_p_ effect size and Greenhouse Geisser (GG) ε correction factors, where applicable.

### Data Availability

The datasets generated and/or analyzed during the current study are available on request (please send an e-mail to: eleonore.smalle@uclouvain.be).

### Ethical approval and informed consent

Participants received financial compensation at the end of the experiment (£10/hour). All participants gave their written informed consent prior to the study. The procedure was approved by the Central University Research Ethics Committee (CUREC) at the University of Oxford (Reference: R45415/RE001). The methods were care carried out in accordance with the standard TMS guidelines and regulations.
